# Minimizing normal tissue low dose bath for left breast Volumetric Modulated Arc Therapy (VMAT) using jaw offset

**DOI:** 10.1002/acm2.14365

**Published:** 2024-05-17

**Authors:** Yongqian Zhang, Weihua Fu, Edward Brandner, Sharon Percinsky, Mary Moran, M. Saiful Huq

**Affiliations:** ^1^ Department of Radiation Oncology University of Pittsburgh School of Medicine and UPMC Hillman Cancer Center Pittsburgh Pennsylvania USA

**Keywords:** breast cancer, low‐dose bath effect, Volumetric Modulated Arc Therapy (VMAT)

## Abstract

**Purpose:**

With proper beam setup and optimization constraints in the treatment planning system, volumetric modulated arc therapy (VMAT) can improve target dose coverage and conformity while reducing doses to adjacent structures for whole breast radiation therapy. However, the low‐dose bath effect on critical structures, especially the heart and the ipsilateral lung, remains a concern. In this study, we present a VMAT technique with the jaw offset VMAT (JO‐VMAT) to reduce the leakage and scatter doses to critical structures for whole breast radiation therapy.

**Materials and methods:**

The data of 10 left breast cancer patients were retrospectively used for this study. CT images were acquired on a CT scanner (GE, Discovery) with the deep‐inspiration breath hold (DIBH) technique. The planning target volumes (PTVs) and the normal structures (the lungs, the heart, and the contralateral breast) were contoured on the DIBH scan. A 3D field‐in‐field plan (3D‐FiF), a tangential VMAT (tVMAT) plan, and a JO‐VMAT plan were created with the Eclipse treatment planning system. An arc treatment field with the x‐jaw closed across the central axis creates a donut‐shaped high‐dose distribution and a cylinder‐shaped low‐dose volume along the central axis of gantry rotation. Applying this setup with proper multi‐leaf collimator (MLC) modulation, the optimized plan potentially can provide sufficient target coverage and reduce unnecessary irradiation to critical structures. The JO‐VMAT plans involve 5–6 tangential arcs (3 clockwise arcs and 2–3 counterclockwise arcs) with jaw offsets. The plans were optimized with objective functions specified to achieve PTV dose coverage and homogeneity; For organs at risk (OARs), objective functions were specified individually for each patient to accomplish the best achievable treatment plan. For tVMAT plans, optimization constraints were kept the same except that the jaw offset was removed from the initial beam setup. The dose volume histogram (DVH) parameters were generated for dosimetric evaluation of PTV and OARs.

**Results:**

The D_95%_ to the PTV was greater than the prescription dose of 42.56 Gy for all the plans. With both VMAT techniques, the PTV conformity index (CI) was statistically improved from 0.62 (3D‐FiF) to 0.83 for tVMAT and 0.84 for JO‐VMAT plans. The difference in the homogeneity index (HI) was not significant. The D_max_ to the heart was reduced from 12.15 Gy for 3D‐FiF to 8.26 Gy for tVMAT and 7.20 Gy for JO‐VMAT plans. However, a low‐dose bath effect was observed with tVMAT plans to all the critical structures including the lungs, the heart, and the contralateral breast. With JO‐VMAT, the V_5Gy_ and V_2Gy_ of the heart were reduced by 32.7% and 15.4% compared to 3D‐FiF plans. Significantly, the ipsilateral lung showed a reduction in mean dose (4.65–3.44 Gy) and low dose parameters (23.4% reduction for V_5Gy_ and 10.7% reduction for V_2Gy_) for JO‐VMAT plans compared to the 3D‐FiF plans. The V_2Gy_ dose to the contralateral lung and breast was minimal with JO‐VMAT techniques.

**Conclusion:**

A JO‐VMAT technique was evaluated in this study and compared with 3D‐FiF and tVMAT techniques. Our results showed that the JO‐VMAT technique can achieve clinically comparable coverage and homogeneity and significantly improve dose conformity within PTV. Additionally, JO‐VMAT eliminated the low‐dose bath effect at all OARs evaluation metrics including the ipsilateral/contralateral lung, the heart, and the contralateral breast compared to 3D‐FiF and tVMAT. This technique is feasible for the whole breast radiation therapy of left breast cancers.

## 1 BACKGROUND

Numerous randomized data and meta‐analyses showed that adjuvant radiation therapy significantly reduced the risk of local recurrences and improved long‐term survival in breast cancer patients.^1^, ^2^ In addition to improving the local and regional control, radiation therapy after lumpectomy can improve cosmetic outcomes and quality of life compared to mastectomy.^3^ Meanwhile, radiation therapy after mastectomy also provides benefits including preserving breast appearance and maintaining quality of life.^4^, ^5^

3D‐conventional irradiation (3D‐CRT) and the 3D field‐in‐field (3D‐FiF) techniques have been the most traditional techniques for the treatment of breast cancer because of their relatively simple setup and high treatment efficiency.^6^, ^7^, ^8^, ^9^ With a 3D approach, target coverage is compromised due to dose constraints to normal structures in some cases. Planning limitations for whole breast radiotherapy were related to a lack of dose conformity and inhomogeneity due to the continuous change in the 3D breast shape and the low density of the lung tissues.^10^ The inhomogeneity in the PTV volume and the undesired doses to the skin and adjacent organs may result in acute and long‐term toxicities.^11^, ^12^

VMAT has been shown in several studies to improve dose coverage and homogeneity within the PTV compared to conventional techniques in breast cancer treatment.^13^, ^14^, ^15^, ^16^, ^17^, ^18^, ^19^, ^20^ Fogliata et al used avoidance sectors on the full arc trajectory for whole breast irradiation. This method reduced the mean doses to all critical structures but increased the high‐dose spillage in the surrounding healthy tissue. The shortened arc angles also resulted in higher skin dose.^16^ Pasler's study showed that the tangential VMAT technique provided better OAR sparing with the cost of target coverage and homogeneity.^17^ Similar to tangential 3D plans, tangential VMAT refers to using small partial arcs or arcs with avoidance sectors to deliver a base portion of the dose, reducing the doses to the lungs, heart, and other critical structures. Kuo et al utilized five partial arcs to produce excellent PTV coverage and homogeneity with acceptable normal tissue exposure.^18^ Yu et al. presented an innovative tangential VMAT technique to provide sufficient target coverage and homogeneity while reducing the mean doses to critical structures.^19^ However, the limitation of tangential‐based VMAT in breast cancer treatment was the potential for increased low‐dose irradiation to critical structures surrounding the target area. This is known as the “low‐dose bath” and can lead to an increased risk of developing secondary cancers in these healthy tissues or undesirable acute side effects.^20^, ^21^

Some hybrid techniques have been presented by combining multiple radiation therapy delivery methods to limit low‐dose baths to critical structures while maintaining target coverage. Jöst combined IMRT and VMAT to reduce the doses to the heart and ipsilateral lung.^22^ Stanton et al.^23^ used an iterative knowledge‐based VMAT planning model to reduce the low‐dose bath effect. Zhang^24^ combined bolus electron conformal therapy with intensity modulated radiotherapy (IMRT) and VMAT techniques for breast cancer treatment. This hybrid planning strategy produced clinically acceptable target coverage and reduced OAR doses. Xie compared non‐coplanar VMAT (NC‐VMAT) plans with various radiation therapy techniques and concluded that NC‐VMAT and multiple arc VMAT produced lower radiation exposure to critical structures.^25^

The aim of this study was to present a VMAT technique with the jaw offset (JO‐VMAT) to reduce the leakage and scatter doses to critical structures for whole breast radiation therapy. The dosimetric performance of the JO‐VMAT was evaluated and compared with 3D‐FiF and conventional tangential VMAT plans. The performance of the JO‐VMAT on normal structure sparing might encourage the clinical implementation of the technique for the treatment of breast cancer.

## 2 MATERIALS AND METHODS

Ten patients with left breast cancer were retrospectively selected for this study. The patients had been treated with 3D‐FiF plans using the deep‐inspiration breath hold (DIBH) technique. The clinical target volume (CTV) was delineated based on the RTOG guidelines.^26^ A 5 mm margin was added to the CTV to generate the PTV. A PTV1_Eval was created by excluding from the breast PTV the bony thorax and lung (anterior rib surface) and 5 mm from the skin surface to avoid the build‐up region affecting the PTV coverage.^27^

All plans were generated for a prescription of 42.56 Gy in 16 fractions. The V105% was <10% and the maximum dose was less than 110% of the prescription dose. A tangential VMAT plan (tVMAT) and a JO‐VMAT plan were created with the Eclipse treatment planning system (Varian, Palo Alto, USA) on a TrueBeam Linac (Varian, Palo Alto, USA) equipped with a high‐definition multi‐leaf collimator (2.5 mm leaf width in the central region of 8.0 cm and 5 mm spatial resolution in the periphery).

### 2.1 3D‐FiF

The 3D‐FiF plans were generated manually with the forward planning method. A pair of 6‐MV tangential fields were first selected to provide PTV dose coverage while minimizing exposures to adjacent OARs. Collimator angles of 0°−15° were used to reduce scatter and Multi‐Leaf Collimator (MLC) leakage dose to normal structures, depending on the breast contours and setup incline angles. The dose distribution was calculated and projected to the beam's‐eye‐view (BEV). The subfields with mixed energies were added and shaped with MLCs to shield the areas receiving doses >107% of the prescription dose. A total of 4−6 subfields were added to each plan and the weightings were adjusted to improve the dose homogeneity within the target volume. The open tangential beams contributed the majority dose to the PTV (75%–90% of total MUs) and the subfields contributed the remainder.

### 2.2 VMAT planning

**FIGURE 1:**
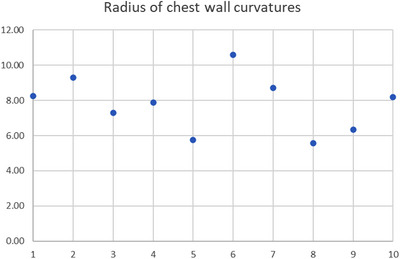
Radius of chest wall curvatures at the center of the breast for the 10 patients with a median value of 8.0 cm (ranging from 5.57 to 10.60 cm).

The curvature of the inner chest wall can vary from person to person, but the basic conical shape is generally consistent. In this study, we calculated the radius of the chest wall curvature in the transversal plane at the center of the breast with the least squares method for all 10 patients. The results are shown in Figure 1. The median curvature radius of the inner chest wall was 8.0 cm (ranging from 5.57 to 10.60 cm).

**FIGURE 2:**
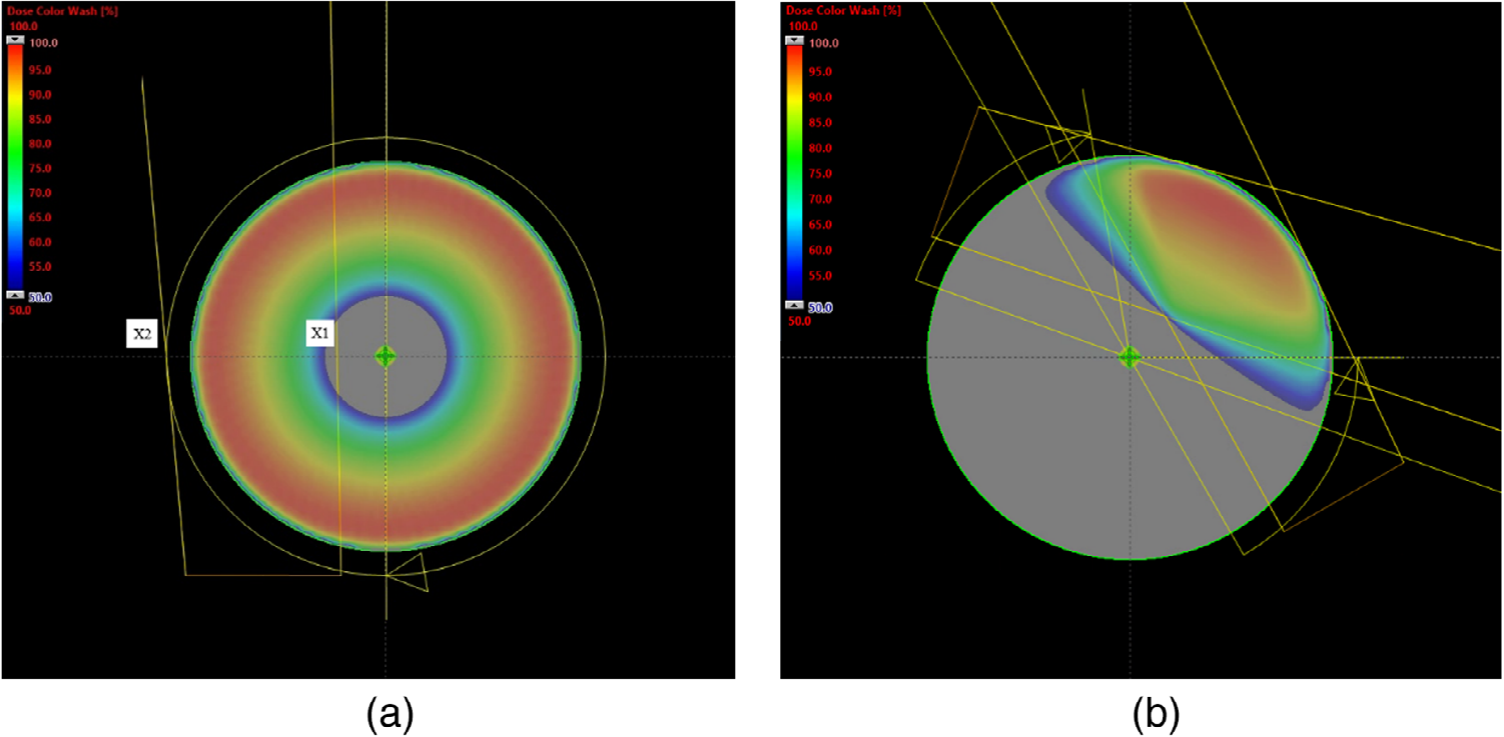
Illustration of the jaw offset for an arc treatment field on a 16 cm diameter cylinder phantom. (a) For a clockwise arc, the X1 jaw position was fixed to be 2 cm across the central axis and the X2 jaw was fixed to be 9 cm with a full rotation of the gantry; For a counterclockwise arc, the X2 jaw position was fixed to be 2 cm across the central axis and the X1 jaw was 9 cm with a full rotation of the gantry. The resultant 50% isodose line was about 2.4 cm from the isocenter whereas the 80% isodose line was 4 cm from the isocenter. A JO‐VMAT plan without MLC modulation on the phantom was shown in (b), and the resultant dose distribution was displayed. The high dose region was about 4 cm from the isocenter.

For illustrative purposes, the representative dose distribution of an arc beam with the jaw offset is shown in Figure 2. A 16 cm diameter cylinder phantom was used to calculate the dose distribution and the isocenter was placed at the center of the phantom. With the clockwise arc, the X1 jaw was fixed to be 2 cm across the central axis and the X2 jaw was fixed to be 9 cm for a full rotation of the gantry (180.1° to 179.9°); For counterclockwise arcs, the X2 jaw was fixed to be 2 cm offset and the X1 jaw was 9 cm for a full rotation of the gantry (179.9° to 180.1°, see Figure 2a). The resultant 50% isodose line was about 2.4 cm from the isocenter whereas the 80% isodose line was 4 cm from the isocenter. This setup generated a donut‐shaped high dose distribution and a cylinder‐shaped low dose volume along the central axis of gantry rotation. A JO‐VMAT plan without MLC modulation on the phantom was shown in Figure 2b, and the resultant dose distribution was displayed. The high dose region was about 4 cm from the isocenter. Applying this setup for breast treatment planning with proper MLC modulation, the optimized plan potentially can provide acceptable target coverage and reduce the low‐dose bath effect on critical structures.

The JO‐VMAT plans were designed with 5−6 tangential arcs (3 clockwise arcs and 2−3 counterclockwise arcs) with the beam span of 50° to 60°. As can be seen the high dose region was about 4 cm from the isocenter in Figure 2a,b, the isocenter of the JO‐VMAT plan was moved to the lung, 3–4 cm from the chest wall so that the target was placed in the high dose cloud. The medial arcs typically start from around 330° to avoid direct irradiation to the contralateral breast and stop at around 30° to spare posterior tissues. The lateral arcs typically start from 140° and stop at around 80° to avoid direct irradiation to the contralateral breast and the lung. The Arc angles were adjusted for patient‐specific anatomy or OARs as required and the collimator angle was also adjusted to make the offset jaw trajectory conform to the curve of the chest wall. The VMAT plans were optimized with objective functions specified to achieve PTV coverage and homogeneity. For OARs, objective functions were specified individually for each patient to achieve the best treatment plan. The heart and the contralateral breast were set up as avoidance structures in optimization to reduce the scatter and leakage doses. For tVMAT plans, all optimization constraints remained the same, except for the removal of the jaw offset from the initial beam settings.

### 2.3 Plan evaluation

A dosimetric analysis of each structure was performed by calculating the population mean DVH for each plan type. The conformity index (CI) and the homogeneity index (HI) were used to compare the target dose quality. The plan CI is given by Paddick^28^:

$$\mathrm{CI}=\frac{{\mathrm{TV}}_{\mathrm{PIV}}^{2}}{\mathrm{TV}*\mathrm{PIV}},$$

Where the TV_PIV_ is the target volume covered by the prescription isodose, TV is the target volume and PIV is the body volume covered by the prescription isodose. The HI was defined as

$$\text{HI}=\frac{D_{2\%}-D_{98\%}}{D_{50\%}},$$

Where *D*_2%_ is the minimum dose in 2% of the PTV, D_98%_ is the minimum dose in 98% of the PTV and D_50%_ is the minimum dose in 50% of the PTV.

The OAR doses were determined for the heart (D_max_, D_mean_, V_5Gy_, and V_2Gy_), the lungs (D_mean_, V_30Gy_, V_20Gy_, V_10Gy_, V_5Gy_, and V_2Gy_ and D_max_ to the contralateral lung), and the contralateral breast (D_max_, D_mean_, and V_2Gy_). The results and analyses were based on averaged DVHs among the studied 10 patients. The statistical significance of paired samples was evaluated using a *t*‐Test and the level of statistical significance was set to *p *< 0.05.

## 3 RESULTS

**FIGURE 3:**
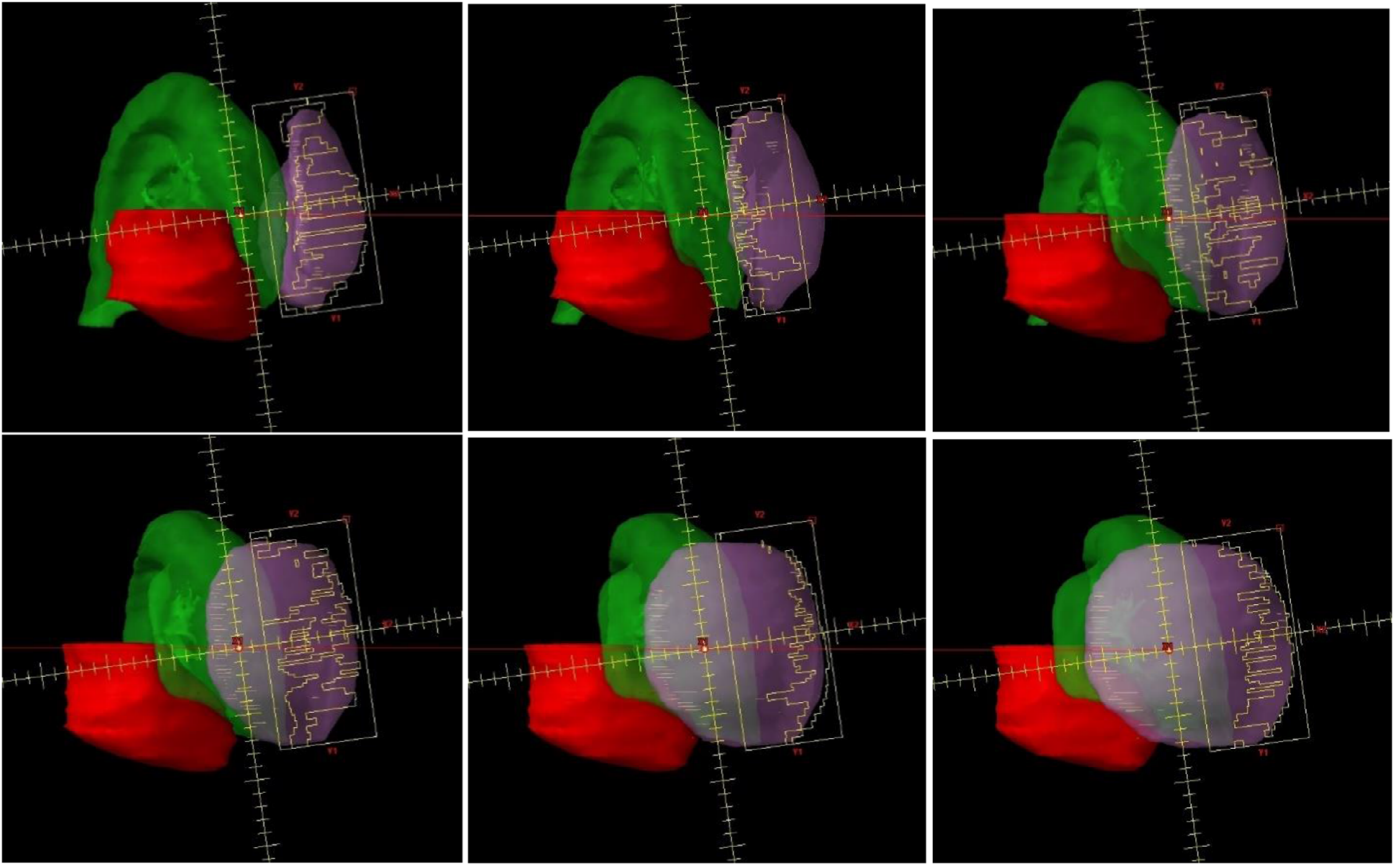
The jaw and MLC trajectories of a JO‐VMAT arc (clockwise) in every 12° of gantry rotation. The arc had a beam span of 60° and the X1 jaw position was −2 cm through the whole arc. The collimator angle was 8° to conform to the curvature of the chest wall. The isocenter was placed in the lung (Green), 3.7 cm from the PTV (Purple). There was no direct irradiation to the heart (Red) as the heart was set as an avoidance structure.

The jaw and MLC trajectories of a JO‐VMAT beam were shown in Figure 3 in every 12° of gantry rotation. The arc had a beam span of 60° (290°−350°) and the collimator angle was 8° conforming to the curve of the chest wall. The setup effectively avoided open fields or closed MLC apertures directed toward the lungs and the heart, thus improved the optimization efficiency, and reduced the MLC leakage and scatter dose to these critical structures.

**FIGURE 4:**
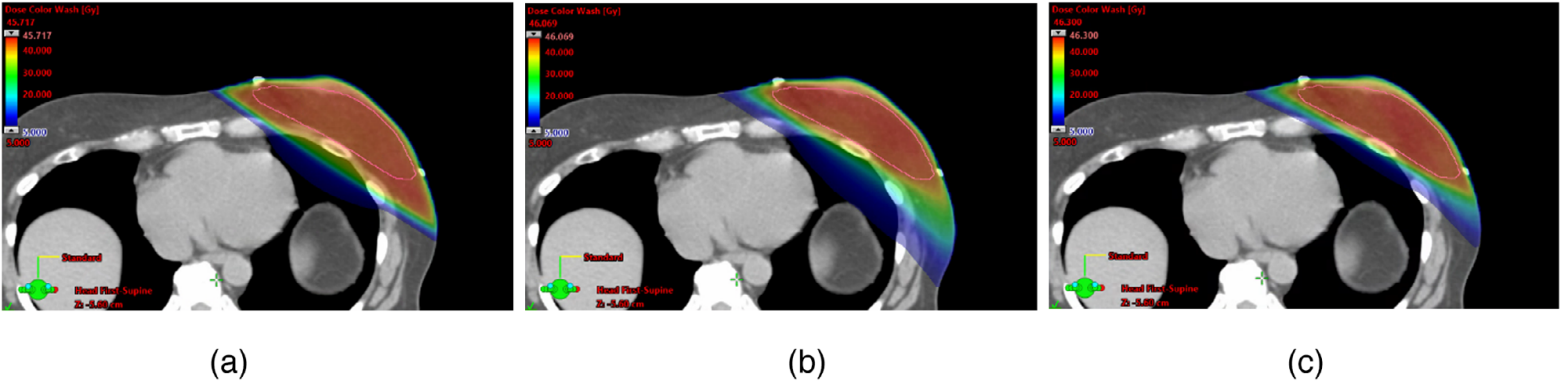
An example dose distribution achieved by (a) 3D‐FiF, (b) tVMAT, and (c) JO‐VMAT techniques. The breast PTV is displayed and dose‐color wash in the axial plane is presented. The JO‐VMAT technique significantly improved the dose conformity to PTV and spared critical structures (the heart and the left lung). Additionally, JO‐VMAT eliminated the low‐dose bath effect at all OARs evaluation metrics including the ipsilateral/contralateral lung, the heart, and the contralateral breast compared to 3D‐FiF and tVMAT techniques.

An example of dose distribution is presented in Figure 4 for the three techniques. All plans were able to meet the PTV dose prescription of D_95%_ > 42.56 Gy. With VMAT, the V _98%_ of PTV was increased from 96.4% to 97.3% on average. Both VMAT techniques achieved significantly higher target dose CI compared to the 3D‐FiF technique (*p *< 0.01). There was no statistical difference in HI among all three techniques. Additionally, JO‐VMAT eliminated the low‐dose bath effect at all OARs evaluation metrics including the ipsilateral/contralateral lung, the heart, and the contralateral breast compared to 3D‐FiF and tVMAT techniques.

**FIGURE 5:**
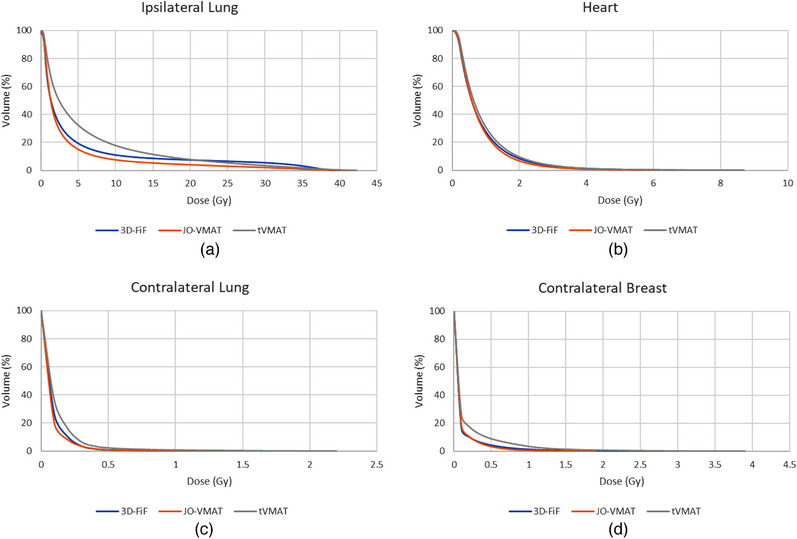
Mean cumulative DVHs (*n* = 10) of the OAR volumes. Heart, contralateral breast, and lungs were shown for 3D‐FiF, JO‐VMAT, and tVMAT techniques studied.

A significant decrease in V_30Gy_ to the ipsilateral lung was observed with both VMAT techniques. However, tVMAT created higher low‐dose volume in critical structures including D_mean_, V_20Gy_, V_10Gy_, V_5Gy_, and V_2Gy_. DVH comparisons (Figure 5) indicated that the JO‐VMAT technique greatly reduced the low‐dose bath to all critical structures in all dosimetric levels. The reduction in D_mean_, V_30Gy_, V_5Gy_, and V_2Gy_ to the ipsilateral lung was 26.0%, 64.2%, 23.4%, and 10.7%, respectively (Table 1).

**TABLE 1:** Dose comparison of the heart, ipsilateral lung, contralateral lung and contralateral breast in 3D‐FiF, tVMAT, and JO‐VMAT treatment plans.

Structure	Dose parameter	3D‐FiF	tVMAT	JO‐VMAT
Heart	D_max_ (Gy)	12.15 ± 6.96	8.26 ± 2.28	7.20 ± 2.83
	D_mean_ (Gy)	0.87 ± 0.16	0.93 ± 0.18	0.82 ± 0.22
	V_5_ (cc)	3.54 ± 5.38	3.74 ± 4.77	2.38 ± 3.78
	V_2_ (cc)	52.43 ± 27.16	62.98 ± 37.83	44.37 ± 40.85
Left lung	D_mean_ (Gy)	4.65 ± 1.18	5.89 ± 1.97	3.44 ± 0.73
	V_30_ (cc)	109.09 ± 52.80	61.13 ± 35.83	39.05 ± 16.60
	V_20_ (cc)	147.76 ± 64.94	148.74 ± 60.18	77.37 ± 24.33
	V_10_ (cc)	217.68 ± 80.53	338.65 ± 157.71	145.63 ± 41.67
	V_5_ (cc)	379.94 ± 111.48	607.85 ± 213.13	291.15 ± 71.45
	V_2_ (cc)	759.49 ± 176.94	1049.35 ± 226.91	678.34 ± 151.44
Right lung	D_max_ (Gy)	1.41 ± 0.64	2.25 ± 1.48	1.24 ± 0.62
	D_mean_ (Gy)	0.07 ± 0.03	0.12 ± 0.05	0.06 ± 0.03
	V_2_ (cc)	0.02 ± 0.04	4.93 ± 10.62	0.02 ± 0.05
Right breast	D_max_ (Gy)	2.54 ± 0.80	4.04 ± 2.33	1.98 ± 0.57
	D_mean_ (Gy)	0.07 ± 0.03	0.16 ± 0.12	0.07 ± 0.03
	V_2_ (cc)	1.68 ± 3.92	8.47 ± 15.41	0.04 ± 0.08

Both VMAT techniques significantly reduced the D_max_ to the heart. The tVMAT technique reduced the heart D_max_ from 12.15 Gy to 8.26 Gy (Table 1). However, the tVMAT increased the V_5Gy_ and V_2Gy_ to the heart from 3.54 ± 5.38cc and 52.43 ± 27.16cc to 3.74 ± 4.77cc and 62.98 ± 37.83cc compared to the 3D‐FiF technique. With the JO‐VMAT technique, the heart D_max_ was reduced to 7.20 Gy, and furthermore, V_5Gy_ and V_2Gy_ volumes were significantly lower compared to 3D‐FiF or tVMAT techniques (*p *< 0.01).

Similar results were observed for the contralateral lung and breast. The tVMAT technique significantly increased the dose to the contralateral lung and breast in terms of D_max_, D_mean_, and V_2Gy_ due to the low‐dose bath effect (Figure 5c,d). JO‐VMAT reduced the D_max_ to the contralateral breast from 2.54 Gy to 1.98 Gy compared to the 3D‐FiF technique; The maximum contralateral lung dose was reduced from 1.41 Gy to 1.24 Gy compared to the 3D‐FiF technique. The reductions in D_mean_ and V_2Gy_ were not significant.

## 4 DISCUSSION

Enormous efforts have been made to reduce the dose to normal structures for the treatment of breast cancer, aiming to minimize the increased risk of secondary cancer induction and acute side effects due to the low‐dose bath effect of VMAT. Mendes's study revealed that the relevant risk of induced cancer from breast irradiation was up to 2.2% considering all thorax organs and Brazilian cancer incidences.^29^ Our data showed that V_5Gy_ and V_2Gy_ to all critical structures were significantly increased with the tangential VMAT setup due to the leakage and scatter doses. Some modified VMAT approaches have been presented by using base‐hybrid plans (Electrons, 3DCRT, IMRT, or VMAT), pre‐defined avoidance sectors/structures, or non‐coplanar beams in order to limit the low‐dose bath.^22^, ^23^, ^24^, ^25^, ^30^, ^31^ The hybrid VMAT planning produced clinically acceptable plans and significantly lowered the dose to the heart and contralateral breast; however, these methods were indeed complex and typically require a certain level of expertise. Additionally, the prolonged treatment time may increase the risk of patient motion potentially degrading the quality and effectiveness of radiation therapy in the treatment of the breast.

In this study, we used predefined avoidance structures to protect the contralateral lung and breast in both tVMAT and JO‐VMAT planning. In Eclipse, avoidance sectors can be used to block unwanted irradiation within the arc rotation regions, and the avoidance structure tool also provides the option to block irradiation completely from entering and/or exiting a specific structure pixel‐by‐pixel in a fluence plane. The data indicated that both VMAT plans provided good coverage (CI and HI of the target) and significantly reduced the D_max_ and high dose volume to the heart and the ipsilateral lung. However, tVMAT could not further reduce the mean dose, V_5Gy_, or V_2Gy_ to the critical structures because of the scatter dose and MLC leakage. With the JO‐VMAT technique, a significant reduction in the low dose volume of the lungs, the heart, and the contralateral breast was achieved at all dosimetric levels.

Dose sparing of the heart is essential since radiotherapy for breast cancer has been linked with increased cardiac diseases. Existing cardiac dose constraints are based on the Qualitative Analysis of Normal Tissue Effects in the Clinic (QUANTEC).^32^ QUANTEC recommended that the V_30Gy_ to the heart should be kept below 46% and mean heart dose less than 15 Gy. On the other hand, the influence of low doses on heart disease has been investigated at the University of Michigan in breast cancer treatment with standard tangential fields.^33^ It was reported that no correlations were found between cardiac doses and changes in perfusion defects, summed stress defect scores, and ejection fractions in the low‐dose region. However, A study by Darby et al. showed that the subsequent rate of ischemic heart disease increased with the cardiac dose in radiation therapy.^34^ The relevant risk of major coronary events was 7.4% per Gray to the mean heart dose. Heart toxicities due to radiation therapy of the breast are a risk, so it is reasonable to keep the heart D_max_, D_mean_, and low dose volume as low as possible. In this study, both VMAT techniques reduced the heart D_max_ with the heart setup as an avoidance structure in the optimization. However, with tVMAT, the mean heart dose, V_5Gy_ and V_2Gy_ were significantly increased due to the low‐dose bath effect. With JO‐VMAT, the D_max_, D_mean_, V_5Gy_, and V_2Gy_ were significantly reduced for all 10 patients compared to the 3D‐FiF or tVMAT plans.

This study focuses exclusively on early‐stage breast cancer patients treated with whole‐breast radiotherapy. Beam setup, avoidance sectors/avoidance structure setup, and optimization strategy will be modified to apply the technique to patients with regional lymph nodes involved. More data will be needed in our future studies to better evaluate the performance of the JO‐VMAT technique.

## 5 CONCLUSIONS

JO‐VMAT technique was evaluated in this study and compared with 3D‐FiF and tVMAT techniques. Our data showed that the JO‐VMAT technique can achieve clinically comparable coverage and homogeneity within PTV and significantly improve the conformity of the treatment plans over 3D‐FiF. Additionally, JO‐VMAT improved the critical structure sparing (the ipsilateral/contralateral lung, the heart, and the contralateral breast) at all dose levels compared to 3D‐FiF and tVMAT plans. This technique is feasible for the whole breast radiation therapy of left breast cancers.

## AUTHOR CONTRIBUTIONS

The authors confirm their contribution to the paper as follows: study conception and design: Yongqian Zhang, Weihua Fu, and Edward Brandner; Treatment planning: Sharon Percinsky, Mary Moran, and Yongqian Zhang; data collection, analysis, and interpretation of results: Yongqian Zhang; draft manuscript preparation: Yongqian Zhang and M. Saiful Huq. All authors reviewed the results and approved the final version of the manuscript.

## CONFLICT OF INTEREST STATEMENT

The authors declare no conflicts of interest.
